# Controlled release of Clenbuterol from a hydroxyapatite carrier for the treatment of Alzheimer’s Disease

**DOI:** 10.1186/s40824-023-00432-4

**Published:** 2023-10-05

**Authors:** Yi-Wen Lin, Chih-Hsiang Fang, Ya-Jyun Liang, Ching-Yun Yang, Wei-Ting Kuo, Feng-Huei Lin

**Affiliations:** 1 Institute of Biomedical Engineering, College of Medicine and College of Engineering, National Taiwan University, No. 1, Sec. 4, Roosevelt Rd, Taipei, 10617 Taiwan; 2 National Taiwan University Hospital, No.7, Chung Shan S. Rd., Zhongzheng Dist, Taipei City, 100225 Taiwan; 3 Division of Biomedical Engineering and Nanomedicine Research, National Health Research Institutes, No. 35, Keyan Road, Zhunan, 35053 Miaoli County Taiwan

**Keywords:** Alzheimer’s disease, Amyloid beta, Clenbuterol, Hydroxyapatite, Controlled release

## Abstract

**Background:**

Alzheimer’s disease is a neurodegenerative disorder, and Aβ aggregation is considered to be the central process implicated in its pathogenesis. Current treatments are faced by challenges such as serious side effects and reduced drug bioavailability. In this study, we developed a drug delivery system for intramuscular injection that uses cellular activity to achieve constant and long-term drug release.

**Methods:**

Synthesized mesoporous hydroxyapatite (SHAP) was prepared via co-precipitation, and hydrophobic surface modification using stearic acid was then used to load clenbuterol by physical absorption, thus creating the drug delivery system. Clenbuterol release was achieved through cellular activity, with macrophage uptake triggering lysosome/endosome disruption, cytoplasmic release, extracellular exocytosis, and subsequent systemic circulation.

**Results:**

We found that clenbuterol-loaded SHAP enabled sustained release for more than 2 weeks and effectively modulated inflammation, reduced Aβ oligomer-induced toxicity, and prevented Aβ aggregation.

**Conclusions:**

Our findings suggest that treatment with clenbuterol loaded in this SHAP delivery system could be a promising strategy for treating Alzheimer’s disease.

**Graphical Abstract:**

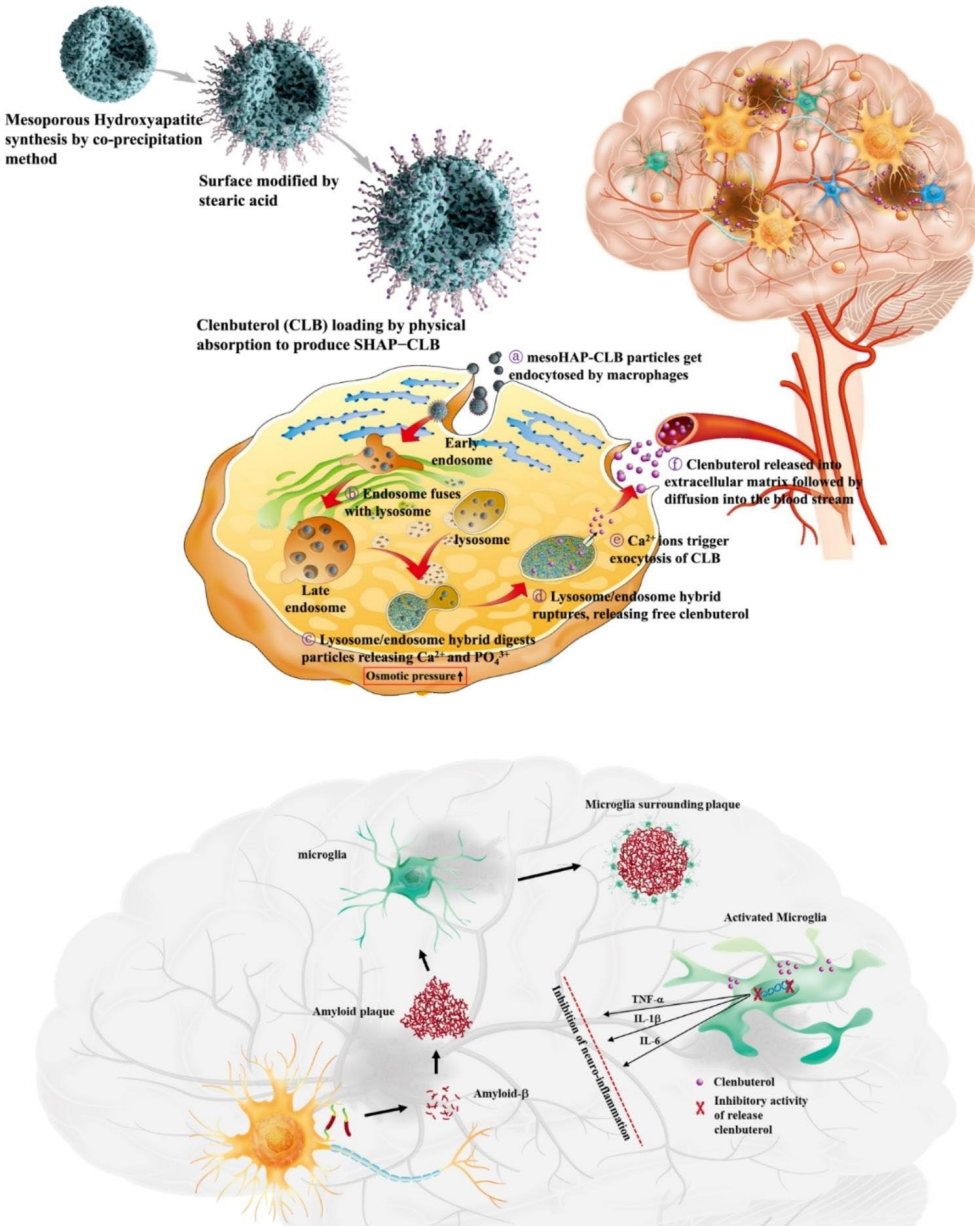

**Supplementary Information:**

The online version contains supplementary material available at 10.1186/s40824-023-00432-4.

## Background

Alzheimer’s disease (AD) is a progressive neurodegenerative disorder. An important neuropathological feature of AD is the accumulation of extracellular amyloid plaques [1] primarily composed of 40 and 42 amino acid-long amyloid β peptides (Aβ) [2]. The current model of disease pathogenesis is the “amyloid cascade hypothesis,” according to which amyloid plaques develop upon abnormal cleavage of amyloid precursor protein (APP) by beta and gamma secretases, leading to the formation of Aβ [3]. AD possibly results from the inflammatory response induced by extracellular Aβ deposits, which are later enhanced by tau aggregates; this inflammatory response driven by activated microglia increases over time as the disease progresses.

In another core pathology of AD, sustained activation of the brain’s resident macrophages (microglia) and other immune cells has been demonstrated to exacerbate amyloid and tau pathologies, and may serve as a link in the pathogenesis of the disorder. In addition to Aβ plaques and neurofibrillary tangles, the brains of patients with AD exhibited evidence of a sustained inflammatory response [4]. Aβ serves as a pro-inflammatory agent, activating several inflammatory components. Their accumulation around neurons causes abnormalities in synaptic functioning, disruption of mitochondrial functioning, and decreased levels of neurotransmitters, such as acetylcholine [5]. Microglia are strongly implicated in the pathology and progressive degenerative nature of AD. In the early stages of AD, initial microglial activation may play a protective role, as it leads to Aβ clearance and release of nerve growth factors. However, when Aβ or other toxic products accumulate, pro-inflammatory phenotypes are activated, thereby damaging the neurons [6]. Endogenous stimuli, including aggregated α-synuclein, mutated superoxide dismutase, Aβ, and tau oligomers, exist in the milieu and may persistently activate pro-inflammatory responses and ultimately cause irreversible neuron loss.

Patients with AD and brains from APP transgenic animals display increased levels of inflammatory cytokines and chemokines, including interferon γ (IFNγ), tumor necrosis factor α (TNFα), interleukin 1β (IL-1β), and interleukin 6 (IL-6) [7]. Microglia exist in different phenotypes. The pro-inflammatory M1 state occurs when microglia are activated, for example, after an acute insult, and causes them to release pro-inflammatory cytokines such as TNFα, IL-1, and IL-6. This activation may occur in response to Aβ. The M2 state is non-inflammatory and is associated with the secretion of anti-inflammatory cytokines such as IL-4, IL-10, IL-13, and TNF-β [8]. It is now thought that in AD, a state of chronic inflammation, microglia are “primed,” wherein they show enhanced sensitivity to inflammatory stimuli and are more easily tipped into the pro-inflammatory M1 state [9].

Clenbuterol (CLB) is a brain penetrant β2 adrenoceptor agonist. Evidence indicates that β2 adrenoceptors have anti-inflammatory and neurotrophic properties that can be neuroprotective [10]—CLB has been shown to have neuroprotective effects in a murine model of motor neuron disease and to enhance cognition in aged rats [11]. Beta 2 adrenergic receptor (β2AR) agonists have considerable anti-inflammatory effects on bone marrow-derived macrophages (BMMs); this was confirmed by the inhibition of phorbol 12-myristate 13-acetate- or liposaccharide (LPS)-induced TNF-α production in rat BMMs as well as diabetes-induced TNF-α production in peripheral blood mononuclear cells [12]. As the β2ARs expressed in the hippocampus and cortex are required for learning and memory [13], CLB is considered a promising potential drug for AD treatment.

**Fig. 1:**
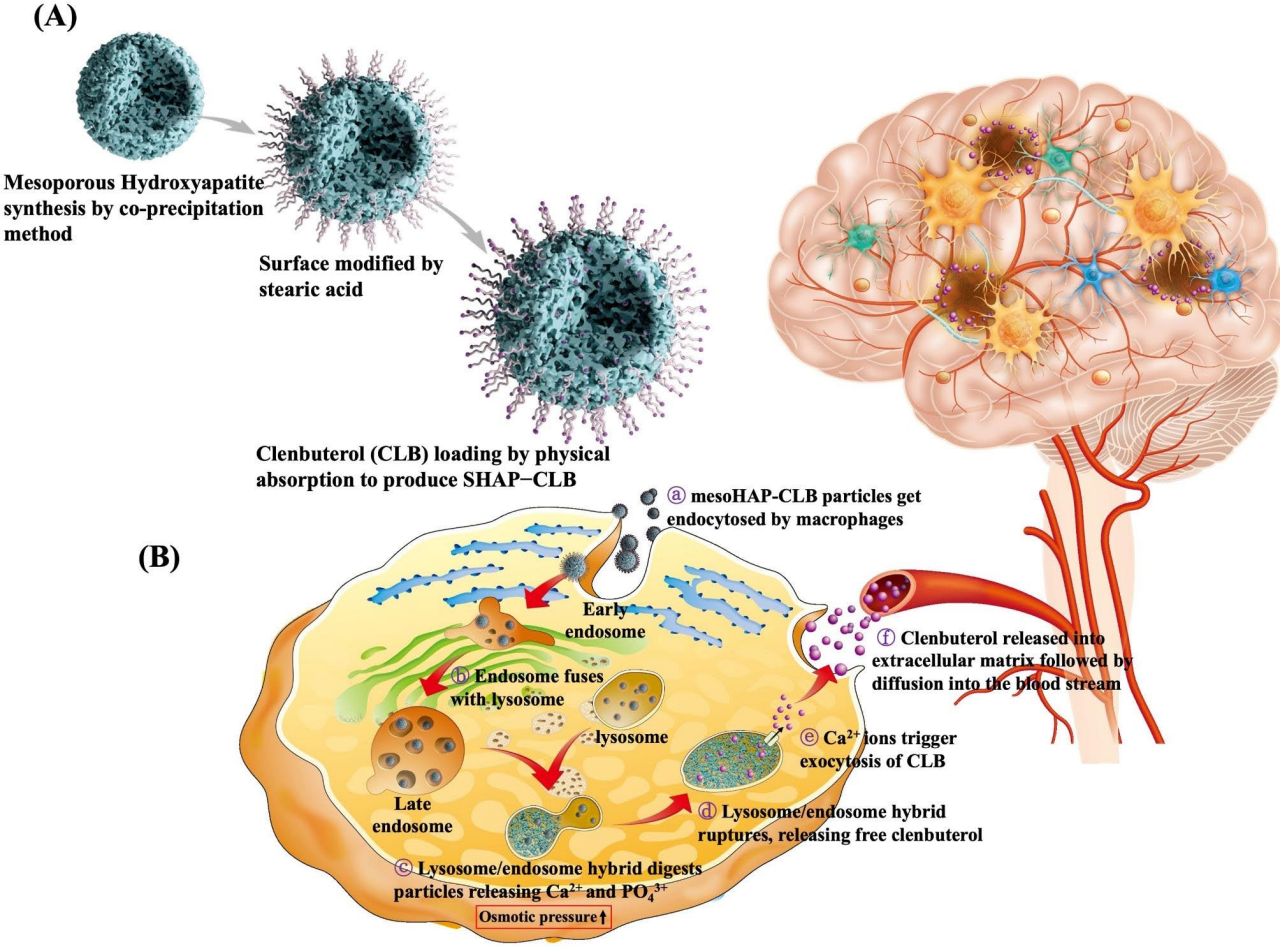
SHAP-CLB preparation and mechanism of drug release. **(A)** First, mesoporous hydroxyapatite (mesoHAP) was synthesized to an appropriate size using a co-precipitation method. Subsequently, several hydrophobic surface modifications were performed using stearic acid to load clenbuterol (CLB) through physical absorption to produce SHAP-CLB. **(B)** Owing to the hydrophobic nature of CLB, it was not efficiently released from SHAP-CLB in an aqueous environment. However, once it was taken up by macrophages, the lysosome/endosome hybrid ruptured as a result of changes in osmotic pressure, leading to the release of CLB into the cytoplasm. CLB was then exocytosis to the extracellular space, where the high concentration of calcium ions (Ca^2+^) facilitated its transfer to the bloodstream. This approach can provide a useful treatment strategy for achieving long-term drug release through a single intramuscular injection, which can solve the problem of nonadherence to medication intake that frequently arises in AD therapy

Development of a drug delivery system that addresses the problem of medication nonadherence due to memory loss is essential. Hydroxyapatite (HAP; Ca_10_(PO_4_)_6_(OH)_2_) is ideal for designing drug carriers because of its excellent biocompatibility and nontoxicity to biological systems [14]. HAP is increasingly being utilized in targeted and controlled drug delivery systems (DDSs), where it demonstrates enhanced adsorption capabilities across biological barriers. By incorporating drugs into porous HAP, it becomes an efficient targeted delivery system to deliver medication. These advancements in drug delivery technology hold promising potential for the development of innovative DDSs, aimed at treating and preventing bone-related disorders like tumors, metastases, and osteoporosis [15]. Additionally, HAP is pH-sensitive and can be degraded into calcium and phosphorus under weakly acidic conditions, rendering it possible to achieve a complete degradation of nanoparticles through pH mediation [16, 17]. In this study, we developed a drug delivery system for intramuscular injection that uses cellular activity to achieve long-term drug release for AD treatment (Fig. 1).

## Methods

### Synthesis of mesoporous HAP

mesoHAP was synthesized using calcium hydroxide (Ca(OH)_2_; Sigma-Aldrich, St. Louis, USA) and phosphoric acid (H_3_PO_4_; Sigma-Aldrich, St. Louis, USA) at a stoichiometric molar ratio (Ca/P molar ratio = 1.67) according to the following chemical reaction: 10 Ca(OH)_2_ + 6 H_3_PO_4_ → Ca_10_(PO_4_)_6_(OH)_2_ + 18 H_2_O. To initiate the reaction, 3.86 g of Ca(OH)_2_ was added in 100 mL of deionized water and heated to 80–85 °C. Subsequently, a stoichiometric amount of 0.3 M H_3_PO_4_ was added dropwise at a rate of 3 mL/min. Egg white was added as foaming agents to facilitate adequate pore size and porosity, and the pH was adjusted to 8.5 with NaOH. The mixture was stirred for 2 h and aged for 20 h at 85 °C. The resulting powder was collected, washed with methanol and deionized water, and calcined at 800 °C to remove albumin, resulting in mesoHAP particles.

### Preparation of hydrophobic CLB

Commercial clenbuterol hydrochloride (CLB·HCl, Sigma-Aldrich C5423, St. Louis, USA) was deprotonated to obtain hydrophobic CLB. Briefly, CLB·HCl was dissolved in dimethyl sulfoxide (DMSO) and incubated overnight with triethylamine (TEA; Sigma-Aldrich, St. Louis, MO, USA) at a molar CLB to TEA ratio of 1:3. The deprotonated CLB was then dialyzed for 24 h to obtain the free base of CLB, which was then subjected to rotary evaporation to remove the residual TEA. After solvent evaporation, the deprotonated CLB powder was collected and stored in a freezer. The quality of deprotonated CLB was evaluated by comparing the main structure with that of CLB·HCl using proton nuclear magnetic resonance [18].

### Surface modification of mesoHAP for hydrophobicity and CLB loading

To modify the surface of mesoHAP for hydrophobicity and CLB loading, stearic acid (Sa) (10% wt relative to the amount of HAP powder) was dissolved in 200 ml deionized water. Subsequently, 2.5 g of HAP powder was added to the Sa solution and the mixture was refluxed at 90 °C for 12 h. The modified HAP powder was collected by centrifugation at 3000 rpm for 10 min, rinsed three times with hot ethanol to remove the free Sa, and lyophilized using a freeze dryer. For drug loading, 50 mg of modified CLB was added to 10 mL of deionized water and mixed with 250 mg of Sa-modified mesoHAP particles. A vacuum system with a rotary pump was used to allow the CLB to enter the meso-HAP pores. The particles were then collected by centrifugation.

### Synthesis and observation of Aβ fibrils

Aβ42 peptides were obtained from Peptide Institute, Inc. (ASIA BIOSCIENCE Co., Ltd., Taiwan) and dissolved in hexafluoro-2-propanol (HFIP; Oakwood Products, Estill, SC, USA) to a final concentration of 1 mM for monomerization. The resulting solution was stored at − 80 °C. The monomerized peptides were resuspended in anhydrous DMSO (catalog number D-2650, Sigma) and diluted to a concentration of 100 µM using Dulbecco’s modified Eagle’s medium (DMEM, Sigma) before use. Aβ fibrils were formed by shaking the solution at 37 °C for 3 days. The morphology of the fibrils, with or without CLB incubation for 3 days, was observed using transmission electron microscopy (TEM). Samples were incubated with 1% uranyl acetate for 1 min before TEM.

### Material characterization and analysis

#### X-ray diffraction analysis of SHAP-CLB

The crystal structures of the synthesized HAP and SHAP-CLB samples were determined using an X-ray powder diffractometer (Rigaku Geigerflex, Tokyo, Japan). The prepared powders were mounted on the sample holder of the diffractometer under Cu Kα with λ = 0.15406 nm radiation and an Ni filter with a potential of 30 kV and current of 15 mA. The specimens were scanned from 10°–70° at a speed of 2°/min. Patterns were analyzed using a model auto-matched to the International Center for Diffraction Database using the Jade 6.0 software by indexing the corresponding peaks using standard diffraction files of apatite (JCPDS #09-0432).

#### Fourier transform infrared analysis of SHAP-CLB

FTIR transmittance spectra were obtained in the 4000–400 cm^− 1^ infrared region (Jasco, FT/IR-4200, Japan) and used to identify the functional groups of HAP, stearic acid, CLB, and SHAP-CLB. The obtained spectra were compared with those of standard KBr and reference materials at a weight ratio of 1:9, and correlation charts were used for a more precise assignment of the observed vibrational peaks.

#### SHAP-CLB morphology assessment

The morphology of the synthesized particles was observed using SEM (Philips XL30, Amsterdam, Netherlands) at an accelerating voltage of 10 kV and a current of 20 mA. The specimens were mounted onto an adhesive copper stub, and gold sputtering was used to increase the imaging resolution and prevent undulations.

### Drug loading capacity and release profile

Thermogravimetric analysis TGA was performed on CLB and SHAP-CLB under a nitrogen atmosphere from 100 to 600 °C at a heating rate of 10 °C/min to evaluate the weight loss regarding the amount of CLB loaded on SHAP during thermal analysis. The release profile of SHAP-CLB was evaluated in saline with a pH value of 7.4 and 3 to mimic the physiological environment and the lysosome/endosome hybrid, respectively. A suspension of 1 g SHAP-CLB in 50 mL of phosphate buffered saline (PBS) was centrifuged at 37 ± 0.5 °C and 100 rpm, and the supernatants were collected and quantified daily for 21 days. The CLB concentration was quantified at 301 nm using a UV spectrometer (V-630; Jasco, Easton, MD, USA) [19]. The efficiency of CLB entrapment in SHAP-CLB was calculated using the following formula:

Entrapment efficiency (%) = (amount of CLB in SHAP/total CLB in the system) × 100% … (1).

### Biocompatibility of SHAP-CLB

The biocompatibility of SHAP-CLB was tested according to the International Standard ISO 10,993 using a WST-1 assay (BIOTOOLS Co., Ltd., Taipei, Taiwan) using the L929 cell line (obtained from the Bioresource Collection and Research Center, Taiwan). The viability of SHAP-CLB cells was evaluated by seeding L929 fibroblasts in 96-well plates at a density of 1 × 10^4^ cells/well and culturing them for 1 day to 80–90% confluence. An extract medium was prepared by incubating 0.2 g/mL HAP, SHAP, and SHAP-CLB for 24 h at 37 °C. The extracted supernatants were collected for subsequent experiments. Zinc diethyldithiocarbamate was used as a positive control and aluminum oxide was used as a negative control. After adding the WST-1 solution to the wells, the plate was incubated for 2 h, and the absorbance of each well was measured at 450 nm using a spectrophotometric plate reader (ELISA reader, Tecan Sunrise, Switzerland). The percentage of viable cells was calculated using the following equation:

Cell viability (%) = ((OD experiment - OD background) ×100)/ ((OD control - OD background))…(2) [20].

### Visualizing SHAP-CLB internalization by RAW264.7 cells

SHAP-CLB internalization by RAW264.7 cells was visualized using TEM and confocal microscopy. The cells were treated with 0.25 mg/mL SHAP-CLB for 24 h and harvested at specific time intervals by centrifugation at 1000 rpm for 5 min. The cell pellets were fixed in 2.5% glutaraldehyde in 0.1 mol L-1 phosphate buffer (pH 7.2) overnight and post-fixed in 1% osmium tetroxide solution for 1–2 h. Subsequently, the cells were rinsed three times with 0.2 M PBS buffer and dehydrated using a series of ethanol solutions (35%, 50%, 70%, 85%, 90%, 95%, and 100%), with each step lasting for 15 min. The cells were cryo-sectioned at the School of Veterinary Medicine of National Taiwan University, and images were captured using TEM (Jeol, JEM-1200EX II, Tokyo, Japan; operated at 100 kV) to observe the internalization of SHAP-CLB by macrophages.

Confocal laser scanning microscopy was used to evaluate the cellular uptake of SHAP-CLB. RAW-264.7 macrophages were seeded in cell imaging dishes containing a 180-µm thick cover glass bottom (ibidi, cat. No. 82,426; Germany). SHAP was labeled with 50 µL fluorescein isothiocyanate (FITC) dye during preparation using hydrophobic association. SHAP was co-incubated with RAW-264.7 cells at a concentration of 0.25 mg/mL for 24 h. To stain the lysosomes, the cells were cultured in a medium containing 75 nM LysoTracker® Deep Red stain for 2 h. For the dual-color detection of SHAP and lysosomes, the cells were resuspended in PBS and fixed with 4% formaldehyde for 10 min. After two washes with PBS, the cells were stained with 100 ng/mL 4′,6-diamidino-2-phenylindole. The cells were excited with alternating 495 nm (FITC) and 647 nm (LysoTracker Deep Red) laser lights. Images were captured using a laser-scanning confocal microscope (LSCM; ZEISS LSM 780) and analyzed.

### Thioflavin T fluorescence assay for monitoring amyloid β aggregation

The progression of amyloid β aggregation was tracked using thioflavin T (ThT), a fluorescent dye originally used to stain amyloid fibrils in histological samples. ThT (Sigma, product no. T3516) was dissolved in PBS and filtered through a 0.2 μm syringe filter. An aliquot of the Aβ stock solution was diluted to 10 µM in pH 7.4 PBS and incubated at 37 °C with shaking. To observe the inhibitory effect of CLB on Aβ aggregation, 10 µM Aβ was mixed with varying concentrations (0.1, 1, 10, and 20 µM) of CLB. Subsequently, 20 µl of 10 µM ThT was mixed with 20 µl of various concentrations of sonicated fibrils to achieve the desired concentrations of ThT and amyloid. ThT fluorescence was measured at 25 °C using a spectrophotometric plate reader (ELISA reader, Tecan Sunrise, Switzerland) with an excitation filter at 450 nm and an emission filter at 482 nm [21], the computation of intensity was performed through the utilization of SoftMax Pro 7.0 software (Molecular Devices, CA, USA). After ThT binds to β-sheet aggregate structures, such as amyloid fibrils, its fluorescence emission changes, allowing the monitoring of amyloid β aggregation.

### Western blot analysis of SHAP-CLB inhibition of LPS-Induced inflammation

The ability of SHAP-CLB to suppress LPS-induced inflammation was evaluated by western blot analysis. LPS (L2880, Sigma) was dissolved in normal saline before injection. BV2 cells were seeded in 6-well plates at a density of 10^5^ cells/well and cultured for 1 day to allow full adhesion. The medium was then supplemented with 20 µM (6.272 µg/mL) SHAP-CLB for 4 h. Subsequently, LPS (1 µg/mL) was added to induce inflammation for 24 h. The medium was then removed and the BV2 cells were washed three times with PBS. The cells were then lysed in 100 µL of ice-cold radioimmunoprecipitation assay buffer, and protein concentrations were quantified using the Bio-Rad protein assay. Equal amounts of proteins were electrophoresed on a 10% sodium dodecyl sulfate-polyacrylamide gel. The separated proteins were transferred onto nitrocellulose membranes and incubated with primary monoclonal mouse antibodies (BIOTOOLS Co., Ltd., Taipei, Taiwan) against IL-6 (1:1000), IL-1β (1:1000), TNF-α (1:1000), and β-actin (1:5000) overnight at 4 °C. Following incubation with the appropriate secondary antibody, the signals were analyzed using an imaging system (Bio-Rad).

### ELISA analysis of SHAP-CLB inhibition of Aβ-induced inflammation

The Aβ oligomer solution was prepared as previously described. BV2 cells were seeded in 16-well plates at a density of 5 × 10^4^ cells/well and cultured for 24 h. Subsequently, the cells were treated with 0.1 µM, 1 µM, 10 µM, or 20 µM of SHAP-CLB in DMEM with 10% fetal bovine serum for 4 h. Then, 10 µM of Aβ oligomer solution or DMEM solution was added to the wells. The medium was collected after 6 h for IL-1β analysis and after 24 h for IL-6 and TNF-α analyses. Cytokine levels were quantified using an ELISA kit (BIOTOOLS Co., Ltd., Taipei, Taiwan), following the manufacturer’s instructions.

### Animal study

The study used 60 male SD rats (BioLASCO, Taiwan; average age, 8 weeks). The study was approved by the Institutional Animal Care and Use Committee (No. 20,190,497) of National Taiwan University. CLB.HCl (Sigma-Aldrich C5423; Lot# BCBQ2163V) powder was stored at 4 °C and diluted in sterile 0.9% saline to a concentration of 20 µM based on the free base before injection. AlCl_3_ (7446-70-0, Sigma-Aldrich) was dissolved in normal saline before injection, and 70 mg/kg body weight of AlCl_3_ was administered intraperitoneally (i.p.) three times weekly for 8 weeks as previously described [22]. The study included five groups: (1) normal rats (negative control); [20] rats treated with PBS (i.p.) [sham]; (3) rats induced with AlCl_3_ (70 mg/kg, i.p.) three times weekly for 8 weeks (positive control); (4) rats induced with AlCl_3_ (70 mg/kg, i.p.) three times weekly for 8 weeks and treated with CLB (6 mg/kg, intramuscular injection) weekly (CLB); (5) Rats induced with AlCl_3_ (70 mg/kg, i.p.) three times weekly for 8 weeks and treated with CLB-loaded SHAP (6 mg/kg, intramuscular injection) weekly [SHAP-CLB]. After 8 weeks, several animal behavioral tests, histological staining, pharmacokinetics, and biochemical analyses were performed.

### Morris water maze (MWM) test

This study used a circular pool partitioned into four quadrants, one of which contained an underwater platform. During each training session, the rat was placed gently in the water from a different drop position and allowed to locate the platform for 2 min. If the rat could not find the platform within this timeframe, it was guided towards it. Once the rat reached the platform, it was allowed to remain there for 30 s. Training was conducted in each quadrant for 4 consecutive days. After four training sessions, the time taken by the rats to reach the escape platform was measured using EthoVision software (Noldus Information Technology, Wageningen, Netherlands). Two phases of retrieval tests were conducted to assess working and spatial memory [23]. To evaluate spatial memory, the time taken by each rat to reach the platform from the starting position was recorded. On the contrary, working memory was assessed by determining the amount of time spent by the rat in the same quadrant (up to 120 s) but without a platform. The time spent in the quadrant without a platform was also recorded [24].

### Functional magnetic resonance imaging (fMRI)

A Bruker Biospec 7T fMRI system (Bruker Corporation, Billerica, Massachusetts, USA) was used for fMRI. Eight weeks after AlCl_3_ administration and treatment, the rats were anesthetized with gas. Two types of images were obtained: anatomical MRI images with T2-weighted rapid acquisition and relaxation enhancement [25] and fMRI images with a single-shot gradient-echo plane (GRE-EPI). The brightness of the hippocampus was measured using ImageJ software after the coronal plane at the center of the hippocampus was photographed. Statistical parameter mapping (SPM) of the hippocampus was performed using the MATLAB SPM software. fMRI data were preprocessed using the analysis of functional neuroimaging (AFNI) toolbox developed by Huang et al. [26]. The brain EPI image of one control rat was selected as the template, and the spatial dimension was used to normalize the brain images of all animals to minimize variations in brain size. After preprocessing, pixel-by-pixel fractional amplitude of low-frequency fluctuations (fALFF) map was calculated using AFNI toolbox, where fALFF was defined as the sum of amplitude across 0.01–0.1 Hz divided by that across the entire frequency range. The hippocampus and whole-brain regions of interest (ROIs) were manually delineated based on the rat brain stereotactic atlas, which are key regions implicated in Alzheimer-related abnormalities according to prior clinical and animal studies [27].

### Pharmacokinetic study of CLB using LC-MS/MS

LC-MS/MS was used to perform quantitative analysis using an LCMS8030 system (Shimadzu, Japan). We used the multiple reaction monitoring mode for mass spectrometric analysis, and the MS/MS parameters were optimized by flow injection analysis. Plasma samples were prepared using simple protein precipitation in methanol. The specificity of the method was tested by analyzing blank rat plasma, blank rat plasma spiked with clenbuterol-d9 (internal standard, IS), and plasma samples collected after CLB administration. A Thermo Hypersil Gold aQ C18 column (50 mm × 2.1 mm) with a particle size of 1.9 μm was used for separation. The mobile phase gradient consisted of (A) 2% methanol in pure water + 0.1% formic acid, and (B) methanol with 0.1% formic acid. The gradient elution was as follows: 30% B (0 min), 30% B (0.5 min), 98% B (2.5 min), 98% B (3.5 min), 30% B (4 min), and 30% B (5.5 min) at a flow rate of 300 µL/min, with a total run time of 5 min. A calibration curve was constructed using the plasma concentration of the quality control (QC) sample and the peak area ratio (y) of the QC sample to the IS. The linear calibration curves of CLB ranged from 0.5 to 200 ng/mL, with correlation coefficients over 0.99952. The injection volume of the final solution was 20 µL for the serum specimens. To evaluate the accuracy, precision, and extraction recovery of the assay, three sequential assays were performed with six replicates of QC samples (5, 50, and 500 ng/mL CLB) for each assay. QC samples were prepared from normal blank rat plasma at concentrations of 0.5, 2, 5, 20, 50, 200, and 500 ng/mL of CLB. The concentrations of the QC samples were calculated based on the calibration curve and compared with those of the control samples with the same concentration. For the brain sample homogenization procedures, a brain sample and an equivalent weight of 25% plasma solution were mixed in a 50 mL centrifuge tube and homogenized completely at 30,000 rpm on ice. The resulting homogenized solution was centrifuged and the supernatant was collected as a test sample. The test sample was filtered through a 0.2 μm GHP filter, and 200 µL of the sample mixture was transferred into a new 96-well 1 mL round collection plate and sealed. Finally, the samples were placed in an autosampler to be injected [28].

### Histological analysis and immunohistochemical staining

Commissioned BIOTOOLS for histological staining (BIOTOOLS Co., Ltd., Taipei, Taiwan). Samples obtained from the different groups were fixed, cut in half, and embedded in paraffin. Sections of 5 µm thickness were prepared and mounted on glass slides. Hematoxylin and eosin (H&E) staining was performed on the slides, and immunohistochemical (IHC) analysis was performed to examine the expression of β-site amyloid precursor protein cleaving enzyme-1 (BACE1). To block endogenous peroxidases, the slides were treated with 0.1% hydrogen peroxide (Sigma-Aldrich, USA) in PBS for 10 min. To prevent non-specific binding, 20 µg/mL of proteinase K (Sigma-Aldrich, USA) solution was added to the slides, which were then incubated for 20 min at 37°C in a humidified chamber. For immunohistochemistry, primary antibodies (BACE1 and amyloid plaques (6E10)) against Tris-buffered saline (TBS) with Aβ [C-Terminal] (25524-1-AP, Proteintech) and 1% bovine serum albumin (BSA) (Abcam, USA) were added to the tissue sections at appropriate dilutions and incubated at 4°C overnight. Sections were washed with TBS containing 0.025% Triton-X 100 with gentle agitation and goat anti-rabbit horseradish peroxidase IgG and 1% BSA were added to the sections. Finally, 3, 3’-diaminobenzidine (DAB; Sigma-Aldrich, USA) substrate solution was used to stain the tissue sections.

### Statistical analysis

The data in this study were obtained from a minimum of three replicates for each data point and presented as the mean ± standard deviation values. Calibration curves were established using known concentrations and with a linear correlation coefficient above 0.99. Statistical significance was determined using one-way analysis of variance with multiple comparison tests, and p-values < 0.05 were considered significant.

## Results

### Characterization of SHAP-CLB

**Fig. 2:**
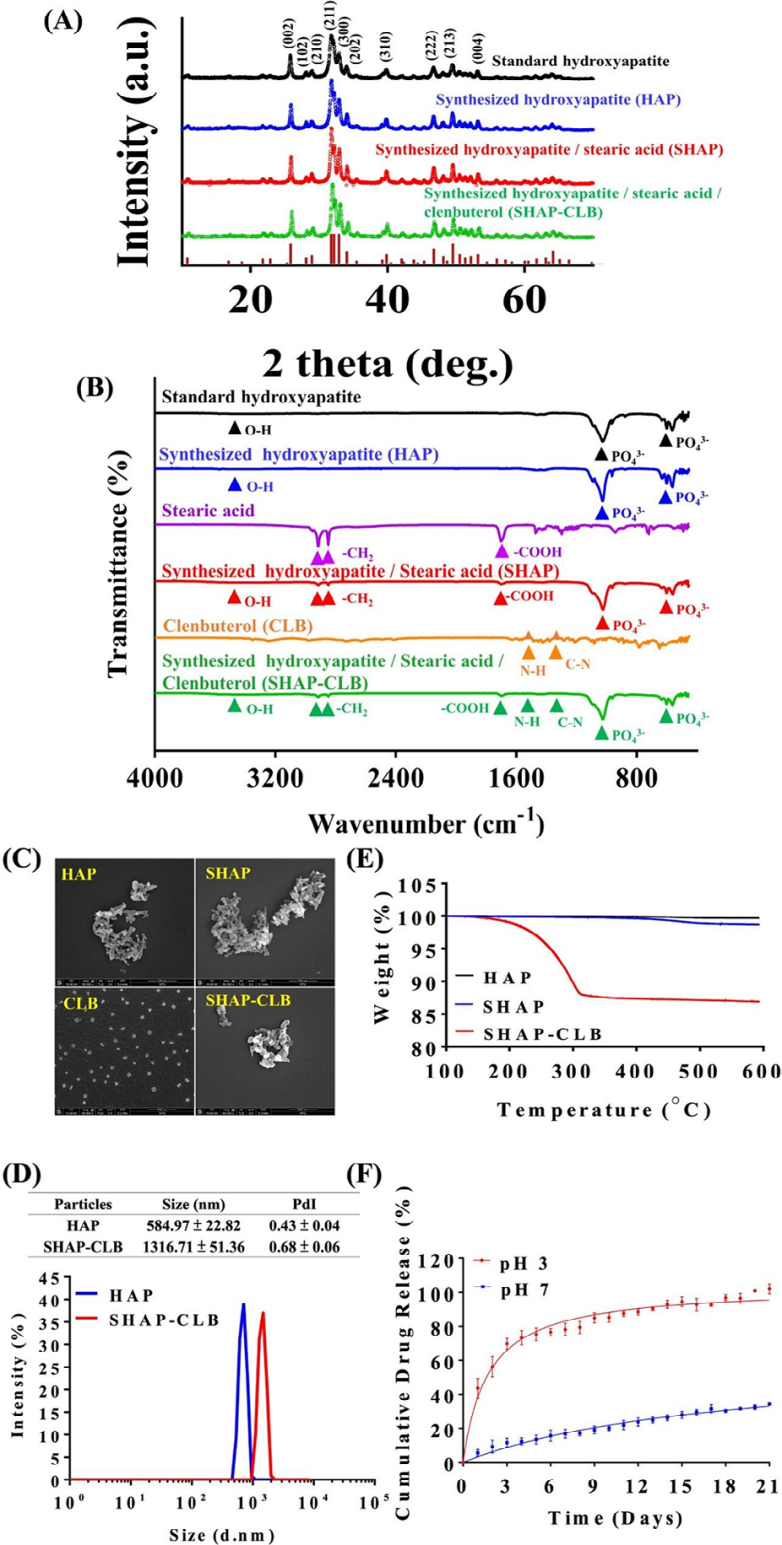
Characterization of SHAP-CLB. **(A)** The X-ray diffractogram of the hydroxyapatite (HAP) sample obtained after using the co-precipitation method was compared with that of the Joint Committee on Powder Diffraction Standards (JCPDS) card number 09-0432 for HA. **(B)** FTIR spectra of HAP, stearic acid, SHAP, CLB, and SHAP-CLB were analyzed. Characteristic absorption bands of HAP were observed in the 600–1100 cm^− 1^ region. Characteristic absorption bands of stearic acid were identified at 1700, 2848, and 2917 cm^− 1^. **(C)** Particle size and morphology. SEM images of SHAP-CLB were captured at 50,000× magnification (scale bar = 500 μm). The images reveal an open and interconnected porous structure for SHAP-CLB. **(D)** The size distribution of SHAP-CLB particles was determined by analyzing DLS data, which indicates that the particle size was within the 500 − 1500 nm range. **(E)** TGA analysis revealed that the weight loss of SHAP-CLB was 13.01% at a temperature of 263.05 ºC, indicating the percentage of CLB loaded in SHAP. **(F)** The drug release profile of SHAP-CLB showed that approximately 12% of CLB was released on the first day at pH 7.4, after which, no further release was observed. However, in an acidic environment (pH 3), SHAP-CLB dissolved and released around 80% of the loaded CLB within 7 days, mimicking the endocytic process of the endosome/lysosome complex

The XRD analysis revealed peaks indicative of HAP (Fig. 2A). The synthesized HAP exhibited characteristic diffraction peaks at 2θ values of 25.9°, 28.1°, 30°, 31.8^o^, 32.2°, 32.9°, 34°, 39.8°, 46.7°, and 49.5° corresponding to (002), (102), (210), (211), (112), (300), (202), (310), (222), and (213), respectively. The X-ray pattern matched the standard HAP pattern of JCPDS No. 09-0432, indicating that the crystal structure was pure HAP and identical to that of the HAP prepared via the precipitation method [29].

Infrared spectra were recorded in the range of 450–4000 cm^− 1^ (Fig. 2B). The diffraction peaks observed at approximately 3500 and 630 cm^− 1^ are characteristic of the stretching and liberation vibrations of the OH groups of the HAP nanoparticles. The diffraction peaks at 1100–900 cm^− 1^ and 610–500 cm^− 1^ were attributed to the vibration of PO_4_^3−^ in the crystalline calcium phosphate phase of HAP. The absorption bands at 567, 604, and 3386 cm^− 1^ were identified as the O − H stretching vibration mode, PO_4_^3−^ bending vibration mode, and PO_4_^3−^ stretching vibrations mode, respectively, as observed for standard HAP. The absorption bands observed at 1361 and 1377 cm^− 1^ corresponded to − COCH_3_. Following modification, the spectrum of stearic acid revealed that the absorption bands observed at 2917, 2848, and 1706 cm^− 1^ corresponded to the C-H and C = O stretching vibration modes, respectively. Additionally, the band observed at 875 cm^− 1^ was assigned to the HPO_4_^2−^ group, which originated from the H + of the COOH group of Sa molecules and the PO_4_^3−^ of HAP nanoparticles. These observations confirmed the interaction between Sa and HAP nanoparticles, indicating that all samples were successfully modified with Sa.

SEM was used to examine the morphology of SHAP-CLB (Fig. 2C). The rod-like grains were aggregated into particles, forming a mesoporous structure with uniform pore size, adequate porosity, and homogeneous distribution. The particle sizes ranged between 80 and 100 nm. These results suggest that the particle size was within the 100 nm–5 μm range suitable for macrophage engulfment [30]. The average particle size and distribution of HAP and SHAP-CLBs were analyzed using dynamic light scattering (DLS), as illustrated in Fig. 2D. The average particle sizes of HAP and SHAP-CLB were determined to be 584.97 and 1316.71 nm, respectively, with polydispersity indices of 0.43 and 0.68, respectively. The size distribution ranged from 0.9 to 3 μm with an average diameter of 1.32 μm owing to aggregations. The average particle size was within the optimal particle size of 0.5–5 μm for cellular endocytosis. The SHAP-CLB particles were expected to be gradually taken up by defense cells to achieve a constant release. The results of the BET analysis are presented in Table 1. The average pore size and porosity of the SHAP-CLB were 253.774 nm and 50.32%, respectively. These values were higher than those of HAP prepared using the traditional method because of the porosity required for better CLB loading efficiency.

TGA was performed to evaluate CLB loading (Fig. 2E). The TGA curve of HAP exhibited a minor weight loss in the temperature range from 25 to 600 °C. The boiling point of stearic acid is 383 °C [31]. The TGA curve of CLB indicated the first weight loss at approximately 100 °C, which could be attributed to the evaporation of surface water, and a second weight loss at 263.05 °C, corresponding to the flashpoint of CLB [32]. The weight loss observed in the synthesized SHAP-CLB represented approximately 13% of the CLB loaded onto the HAP particles. Figure 2 F illustrates the cumulative release of CLB from SHAP-CLB. The release profile of CLB from SHAP-CLB was evaluated in saline with pH values of 7.4 and 3.0, mimicking the physiological environment and the acidic conditions of endosomes/lysosomes, respectively. At pH 7.4, where hydrophobic CLB is generally not released in a water-rich environment, SHAP-CLB exhibited relative stability without significant dissolution. A slight initial burst release of CLB occurred during the first 2 days, possibly due to physical adsorption. No further release of CLB was observed. It is believed that CLB was absorbed and retained within the pores between the HAP nanograins, and may not have been released unless it was exposed to an acidic environment and subsequently dissolved. However, at pH 3.0, HAP-CLB completely dissolved, leading to the release of almost all loaded CLB within 7 days, simulating the endocytic process within the endosome/lysosome complex.

**Table 1:** BET surface area and porosity of SHAP-CLB

Samples	SHAP	SHAP-CLB
BET Surface area (m^2^/g)	23.0854 ± 0.0335	34.7512 ± 0.0847
Correlation Coefficient	0.9999934	0.9999810
Langmuir Surface Area (m^2^/g)	33.8806 ± 0.7257	52.3353 ± 1.2112
Correlation Coefficient	0.998627	0.998397
BJH Adsorption Average Pore Diameter (Å)	268.736	268.502
BJH Desorption Average Pore Diameter (Å)	252.792	253.774

*Brunauer–Emmett–Teller (BET) Surface Area Analysis

*Barrett–Joyner–Halenda (BJH) Pore Size Analysis

### Biocompatibility, cellular uptake, and release mechanism of SHAP-CLB

**Fig. 3:**
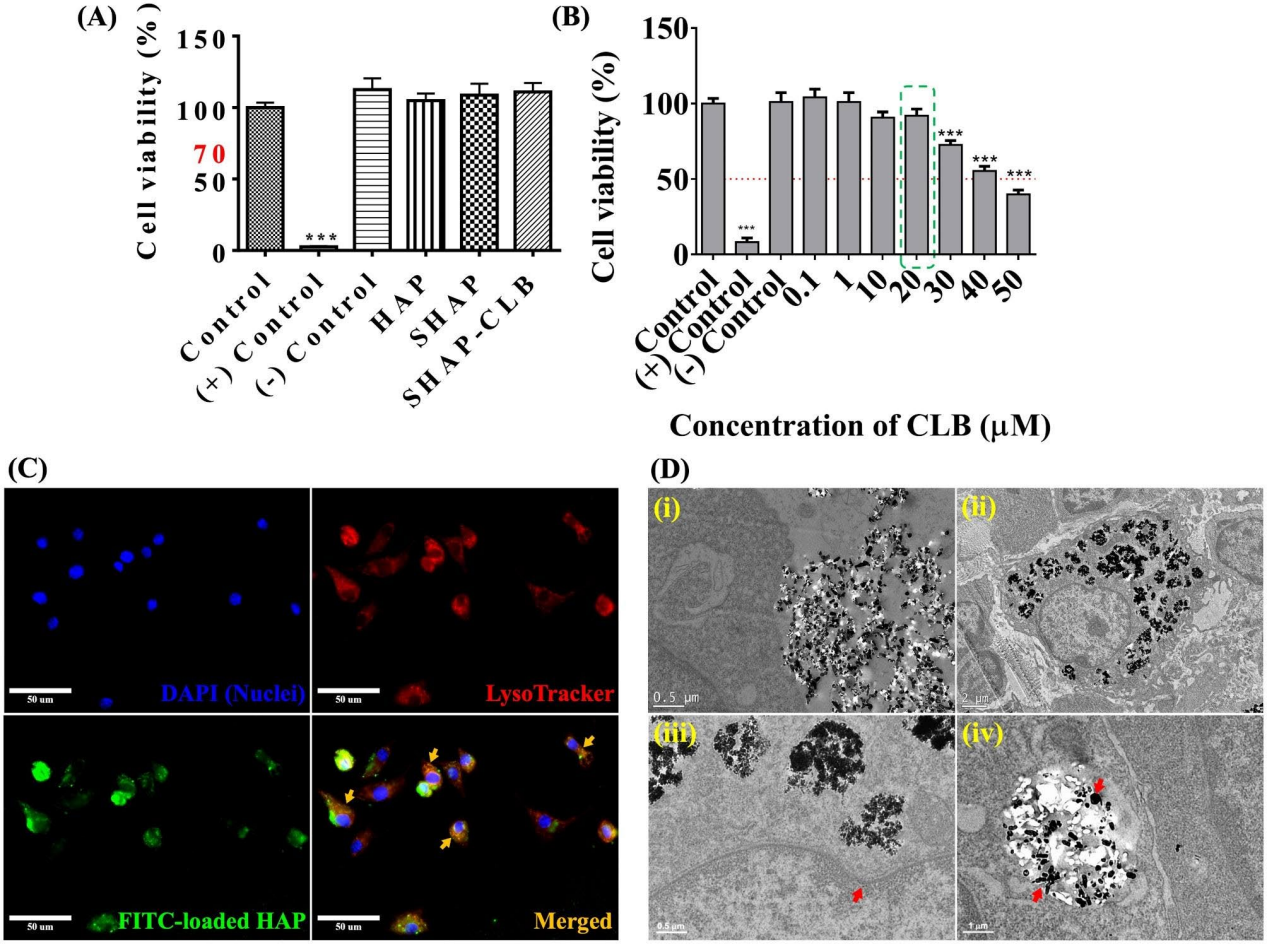
Biocompatibility and mechanism of drug release. **(A)** Viability of L929 cells was determined on day 3 using the WST-1 assay. **(B)** Predicted IC_50_, representing the concentration at which 50% cell (N2a) survival was observed, was determined to be 20 µM for CLB. **(C)** Cellular uptake of FITC-labeled SHAP in RAW264.7 macrophages. Scale bar = 50 μm. **(D)** TEM images of RAW-264.7 cells cultured with SHAP − CLB were obtained to investigate their interactions at the cellular level. (i) The interface between RAW-264.7 cells and SHAP − CLB particles prior to cellular uptake. Scale bar = 0.5 μm. (ii) RAW-264.7 cells can be observed engulfing and enclosing SHAP − CLB particles within endosomes. Scale bar = 2 μm. (iii) The indentation of the cell membrane during the process of endocytosis is depicted in Figure. Scale bar = 0.5 μm. (iv) The SHAP − CLB particles transported to the lysosomes. Scale bar = 1 μm

Cell viability was assessed on day 3, and the findings demonstrated that SHAP-CLB did not exhibit any cytotoxic effects or hinder cell (L929) proliferation. No significant changes were observed compared with the control group (Fig. 3A). According to the ISO-10993-5 guidelines, there were no notable disparities in cell viability among the HAP, CLB, and SHAP-CLB test groups. These observations indicated that the synthesized SHAP-CLB was non-toxic. We initially aimed to determine the relative resistance to CLB in each N2a cell line set. Variations in CLB concentration did not affect the IC_50_ of the drug in the drug-sensitive group (Fig. 3B). The IC_50_ of SHAP-CLB was determined by plotting cell viability against the SHAP-CLB concentration. A concentration of 20 µM CLB was selected as the half-maximal inhibitory concentration for subsequent experiments.

To assess the effectiveness of SHAP in bioimaging experiments, the RAW264.7 macrophages were cultured with FITC-SHAP. After a 24 h incubation period, we observed a significant increase in green fluorescence within the cells, indicating FITC-SHAP (Fig. 3C). Additionally, we noticed clear co-localization between the red fluorescence emitted by LysoTracker, an anisotropic dye, and Cy5 fluorescence. This co-localization strongly suggests that the internalized FITC-SHAP was transported through the endo-lysosomal pathway in RAW264.7 cells (indicated by the yellow arrow), as the LysoTracker fluorescence primarily originates from late endosomes and lysosomes [33]. These observations provide conclusive evidence that SHAP-CLB particles were engulfed by RAW264.7 macrophages through the endocytic process. The release of CLB from SHAP-CLB was dependent on phagocytic activity and was not observed under normal physiological conditions, highlighting the crucial role of intracellular drug release. To investigate the interaction between RAW-264.7 cells and SHAP-CLB particles before endocytosis, TEM images were obtained after a 24 h incubation period (Fig. 3D). Figure 3D-i illustrates the interface between RAW-264.7 cells and SHAP-CLB particles before endocytosis. In Fig. 3D-ii, RAW-264.7 cells internalized SHAP-CLB and enclosed them within endosomes. The enlarged image (Fig. 3D-iii) illustrates the cell membrane indentation during endocytosis. Furthermore, Fig. 3D-iv illustrates the localization of SHAP-CLB particles within lysosomes. Notably, digestion of SHAP-CLB particles occurred within the endosome/lysosome hybrid, and subsequently, the hybrid structure expanded and ruptured owing to changes in osmotic pressure, as indicated by the red arrow.

### Inhibition of Aβ aggregation by CLB

**Fig. 4:**
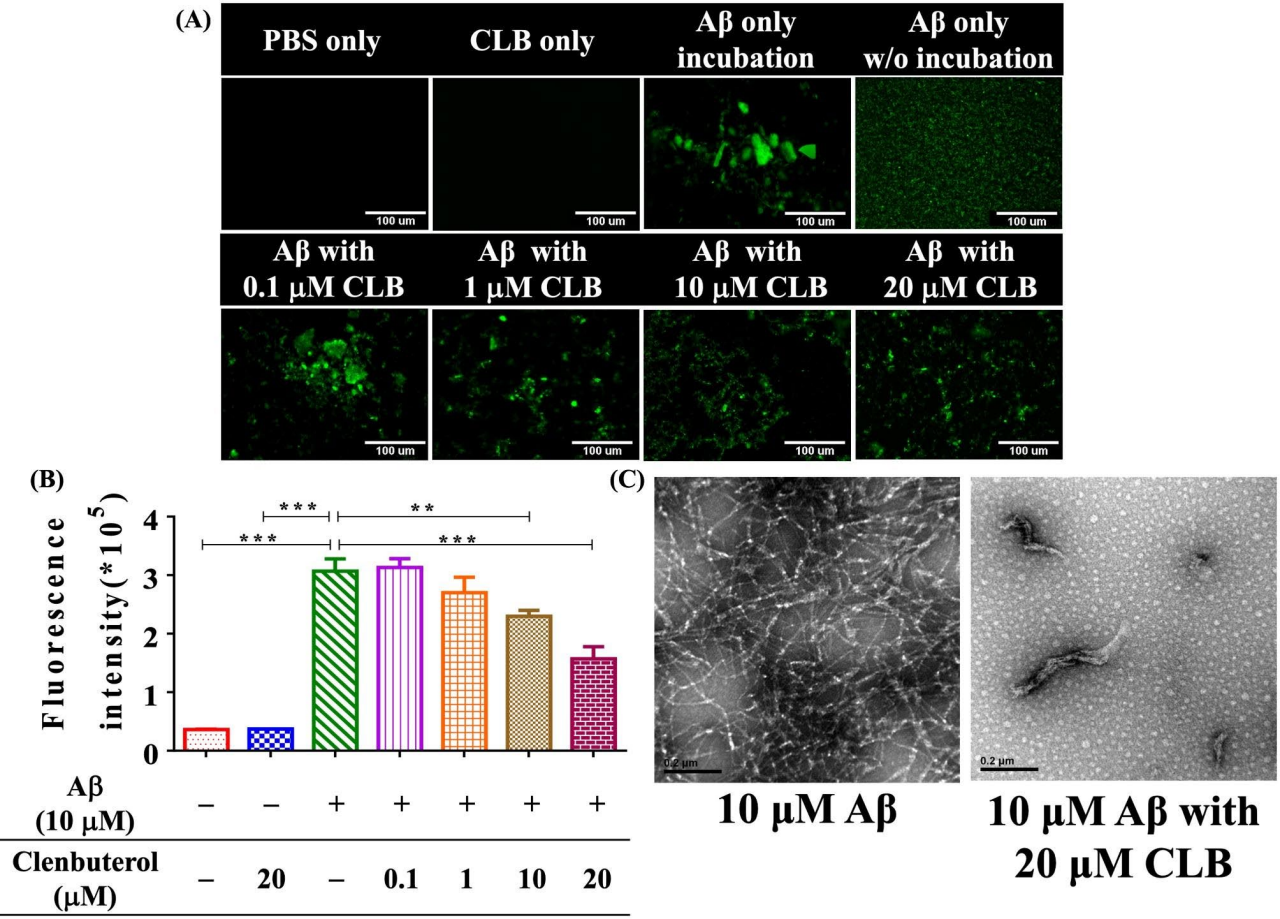
Impact of CLB on the aggregation of Aβ was investigated through various experiments. **(A)** Aβ fibril formation was tracked using ThT fluorescence to measure the fluorescence intensity. **(B)** Fluorescence intensity at different concentrations of CLB was measured and analyzed. The data was obtained from six independent experiments, and statistical analysis revealed significant differences (n = 6, ** P < 0.01 compared with the control, *** P *<* 0.001 compared with the control). **(C)** TEM was used to capture images of negatively stained samples collected at atmospheric pressure

Fluorescence imaging revealed that CLB disentangled amyloid plaques (Fig. 4A) and that the structure of amyloid aggregation became significantly looser with increasing drug concentration (Fig. 4B). The results indicated that incubating Aβ with 20 µM CLB resulted in extensive fragmentation, which was used as the drug concentration in subsequent experiments. TEM images also showed that CLB disassembled amyloid plaques (Fig. 4C), with dense fibrils observed in the absence of CLB. The image on the right was obtained after co-incubating Aβ and CLB for 3 days.

### Inhibitory effect of SHAP-CLB on LPS-induced inflammatory mediators in BV-2 microglial cells

**Fig. 5:**
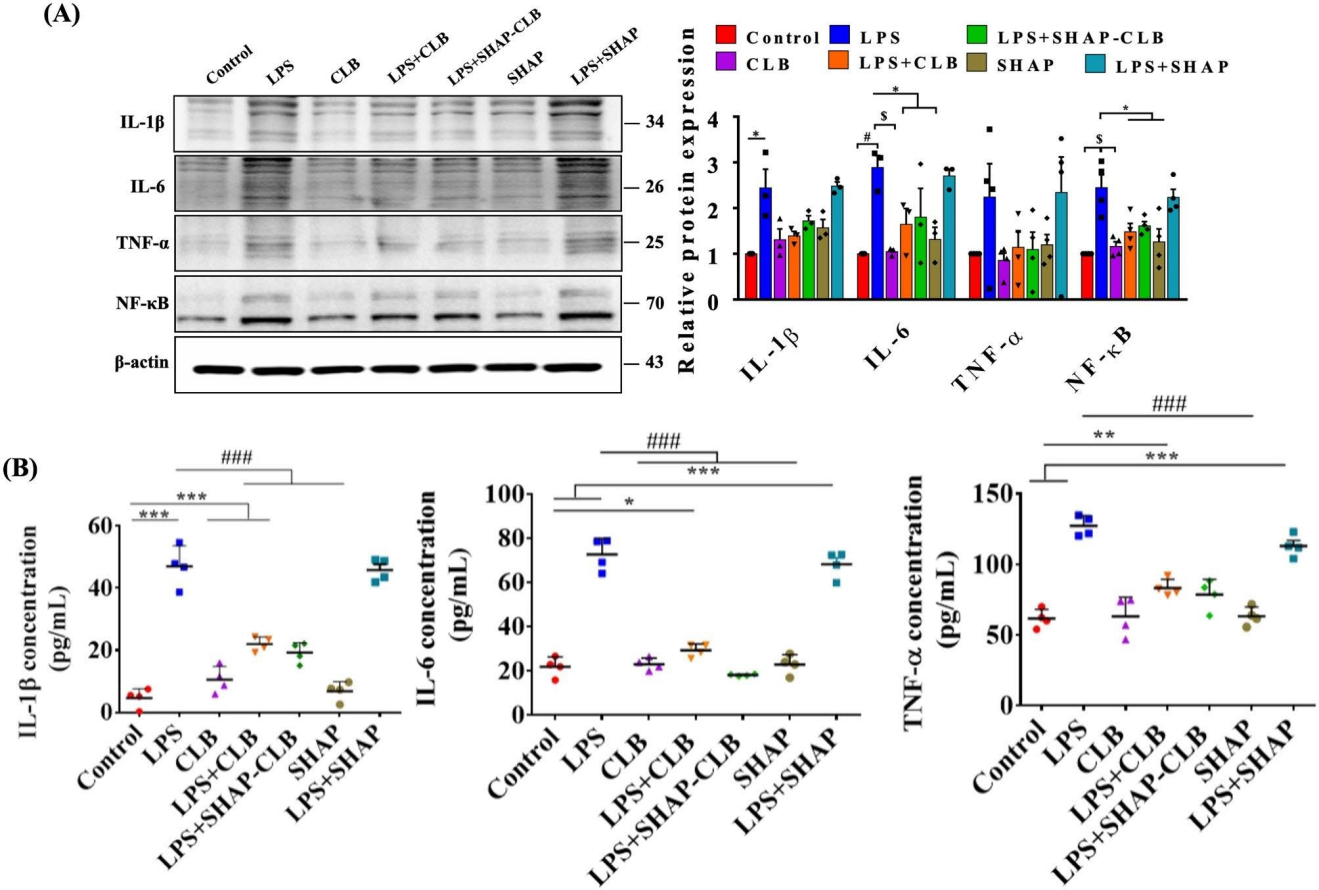
Effects of CLB on LPS-induced protein in BV-2 cells. **(A)** The levels of protein and their secretion were used to measure TNF-α, IL-1β, and IL-6 levels. The relative protein levels were quantified through scanning densitometry and normalized to that of β-actin. The results are presented as the mean ± standard deviation values for each group, derived from three independent experiments. n = 6, *P < 0.05, $P < 0.01, and #P < 0.001. **(B)** The anti-inflammatory effects of SHAP-CLB on Aβ-induced inflammation in BV-2 cells, as determined by the expression analysis of the inflammation-related proteins IL-6, IL-1β, and TNF-α. n = 6, *P < 0.05 versus the control group, **P < 0.01 versus the control group, ***P < 0.001 versus the control group, and ###P < 0.001 versus the LPS-treated group)

The stimulation of BV-2 cells with LPS resulted in a significant increase in the secretion of TNF-α, IL-1β, and IL-6. However, CLB effectively inhibited protein secretion by BV-2 cells. Specifically, treatment of BV-2 cells with LPS alone elevated the production of NF-κB, IL-6, and TNF-α and the expression of IL-1β, compared with the negative control. In contrast, CLB demonstrated significant inhibitory effects on the secretion of IL-1β, IL-6, and TNF-α in LPS-stimulated BV-2 microglial cells, particularly at higher concentrations (20 µM), compared with the LPS-treated control cells. Furthermore, CLB prominently reduced the protein expression levels of IL-1β, IL-6, and TNF-α in LPS-induced BV-2 cells (Fig. 5A). To further validate the anti-inflammatory effects of SHAP-CLB, an ELISA kit was used. BV2 microglial cells were incubated with 10 µM Aβ for 6 h to assess IL-1β levels and for 24 h to assess IL-6 and TNF-α levels. SHAP-CLB effectively mitigated Aβ-induced inflammation in BV2 cells (Fig. 5B). By reducing Aβ-induced inflammation, it is anticipated that neuronal degeneration could be mitigated, thus potentially preventing the development of AD.

### Behavioral experiments and determination of hippocampal activity

**Fig. 6:**
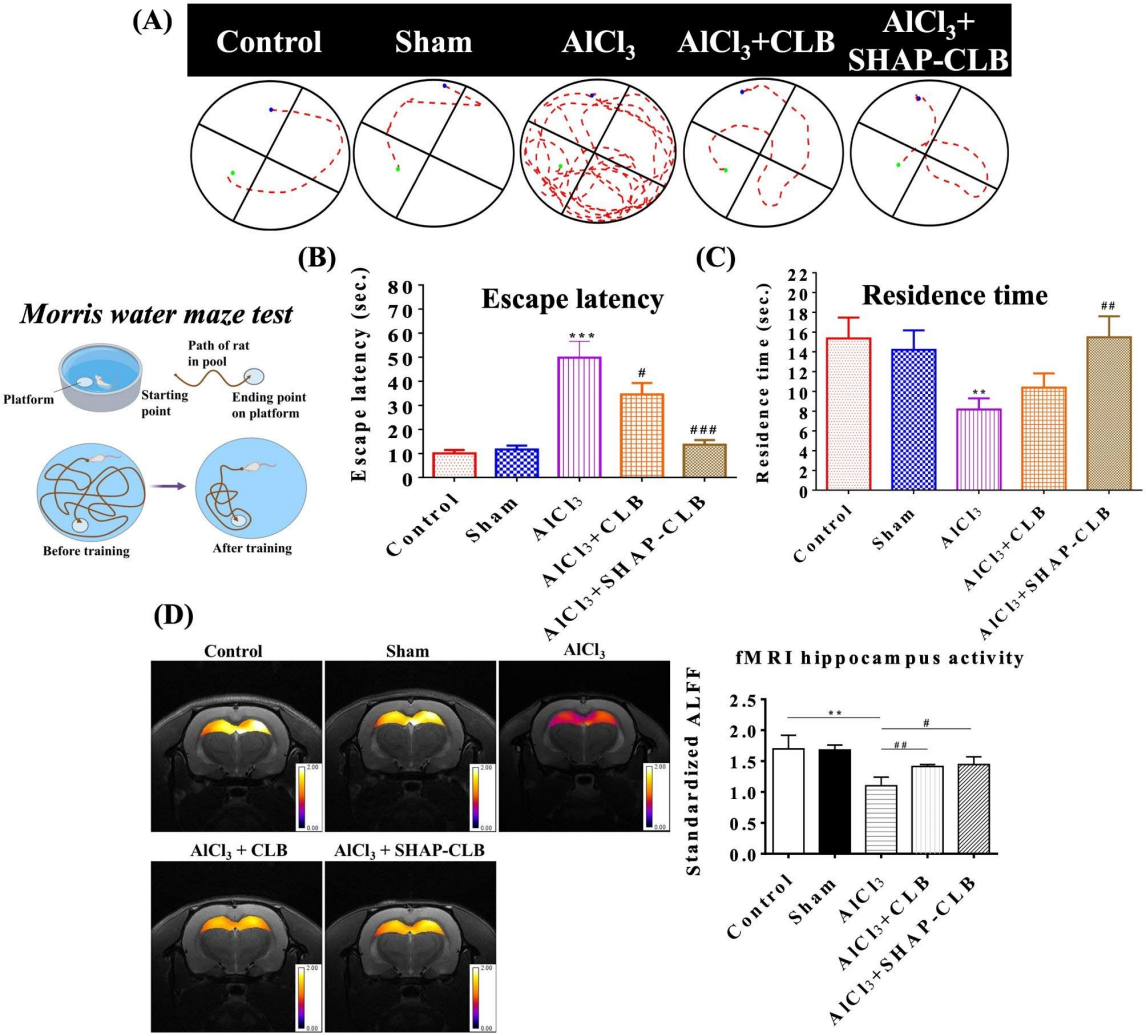
MWM test and hippocampal brain activity. The following groups were used: normal rats (control), rats treated with PBS injection (Sham), rats with AlCl_3_-induced AD (AlCl_3_), rats with AlCl_3_-induced AD treated with CLB (AlCl_3_ + CLB), and rats with AlCl_3_-induced AD treated with SHAP-CLB (AlCl_3_ + SHAP-CLB) (n = 6, *P < 0.05, **P < 0.01, ***P < 0.001 versus control. #P < 0.05, ##P < 0.01, ###P < 0.001 versus the AlCl_3_ group). Hippocampal brain activity was assessed using fMRI analysis. The intensity of brightness within the ROI on single-shot GRE-EPI images was used as a measure of brain activity (n = 6). Compared to that of the control and SHAP-CLB groups, the intensity of brightness within the ROI of the AlCl_3_ group was approximately 15% lower. This decrease in intensity indicates reduced brain activity in the hippocampus in the AlCl_3_ group

The MWM test was used to assess spatial memory in rats with Al-induced AD. Figure 6 A illustrates the swimming paths and escape latencies of the rats. The time taken by the animals to locate the escape platform was quantified. In the SHAP-CLB group, the animals could quickly locate the escape platform in the MWM test, as illustrated in Fig. 6B. Notably, the results of the SHAP-CLB group were significantly different from those of the positive control group. However, the CLB group did not exhibit a performance similar to that of the SHAP-CLB group. The residence time in the quadrant containing the original escape platform was measured to evaluate spatial memory. Figure 6 C illustrates the results obtained for the SHAP-CLB and positive control groups, indicating significant differences between them. A water maze experiment confirmed the effectiveness of SHAP-CLB in the prevention and treatment of AD. The rats in the SHAP-CLB group spent significantly lesser time in the section with the hidden platform than those in the AD group.

rs-fMRI has emerged as a promising method for assessing brain connectivity in patients with AD. In this study, the rats were anesthetized and subjected to fMRI analysis. T2-weighted RARE images were used for anatomical observations, whereas single-shot GRE-EPI images were used to measure brain activity. Coronal MRI images of the hippocampus were acquired, and ROIs were selected. The brightness intensity in the ROI on a single GRE-EPI image was used to determine the brain activity in the hippocampus (Fig. 6D). Functional MRI provided a coronal image of the hippocampus, and an ROI was selected within this region. The Low-Frequency Fluctuation Amplitude (ALFF) index, which reflects brain activity, was calculated based on ROI analysis. The ALFF was significantly lower in the AlCl_3_ group than in the control group, indicating reduced hippocampal brain activity and severe AD. However, following the administration of SHAP-CLB, hippocampal activity significantly increased, showing no significant difference compared with the control group. This suggests that SHAP-CLB treatment restored hippocampal activity to a level comparable to that of the control group.

### Pharmacokinetics of CLB by LC-MS/MS

**Fig. 7:**
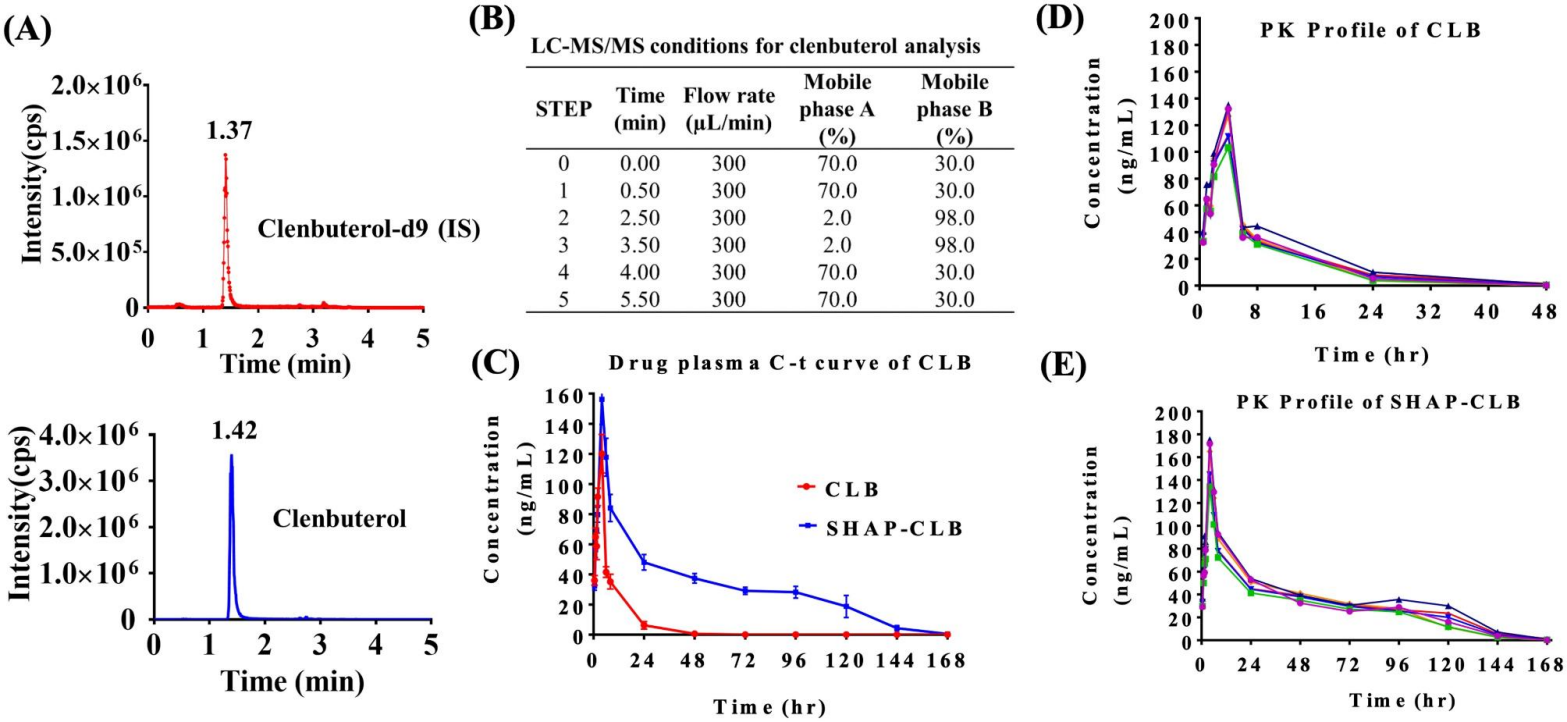
Pharmacokinetic profiles illustrating the mean plasma concentration-time curves of CLB following intramuscular administration of free CLB and SHAP-CLB carrier were examined. Representative LC-MS/MS chromatograms, including **(A)** blank plasma spiked with CLB and IS, and **(B)** LC-MS/MS mobile phase gradient elution condition, are shown. **(C)** The mean plasma concentration-time curves of CLB and SHAP-CLB after intramuscular administration of CLB (6 mg/kg) alone and the carrier of CLB (6 mg/kg). The mean drug plasma concentration-time curves of CLB and SHAP-CLB after intramuscular administration of CLB and drug carriers at a dose of 6 mg/kg are illustrated in **(D)** and **(E)**, respectively (data represent the mean ± standard deviation values, n = 6)

Under the established chromatographic conditions, no interference from endogenous peaks was observed at the retention times of the analytes (Fig. 7). We compared the pharmacokinetic parameters of CLB and SHAP-CLB in each group (Table 2). The mean plasma concentration-time profiles and AUC (0–24 h) are illustrated in Fig. 7. The Cmax values for CLB and SHAP-CLB were 120.185 ± 12.847 ng/mL and 156.240 ± 16.701 ng/mL, respectively. However, CLB was not detectable after 24 h owing to its rapid metabolism. These findings indicate that SHAP-CLB can enhance the stability of drugs in the body and reduce in vivo drug efflux. Measurement of drug content in brain tissues 24 h after intramuscular injection revealed CLB and SHAP-CLB concentrations of 5.24 ± 1.81 ng/mL and 16.08 ± 2.66 ng/mL, respectively (Table 3). In conclusion, we investigated the pharmacokinetics of CLB and SHAP-CLB carriers in rats after multiple intramuscular administrations using LC/MS/MS. Our results indicated no apparent accumulation of CLB following repeated dosing in rats. However, a significant accumulation of SHAP-CLB was observed after multiple-dose administration.

**Table 2:** The pharmacokinetic parameters of LC/MS/MS.

	C_max_ (ng/mL)	T_max_ (hr)	AUC_0 − t_ (ng/mL*hr)	AUC_0 − inf_ (ng/mL*hr)	Time start (hr)	Time end (hr)
CLB	120.19	4.0	4886.43 ± 3744.95	5199.55 ± 4003.23	0.5	168
SHAP-CLB	156.24	4.0	5390.80 ± 4480.44	7361.66 ± 4370.81	0.5	168

**Table 3:** Drug content in brain tissues and brain homogenate obtained by liquid–liquid extraction and subsequently analyzed using LC/MS/MS

	Concentration (ng/mL) 24 h after injection	Concentration (ng/mL) 56 days after AD treatment
CLB	5.24 ± 1.81	N.A.
SHAP-CLB	16.08 ± 2.66	2.38 ± 0.31

### Histological analysis and therapeutic effect of SHAP-CLB

**Fig. 8:**
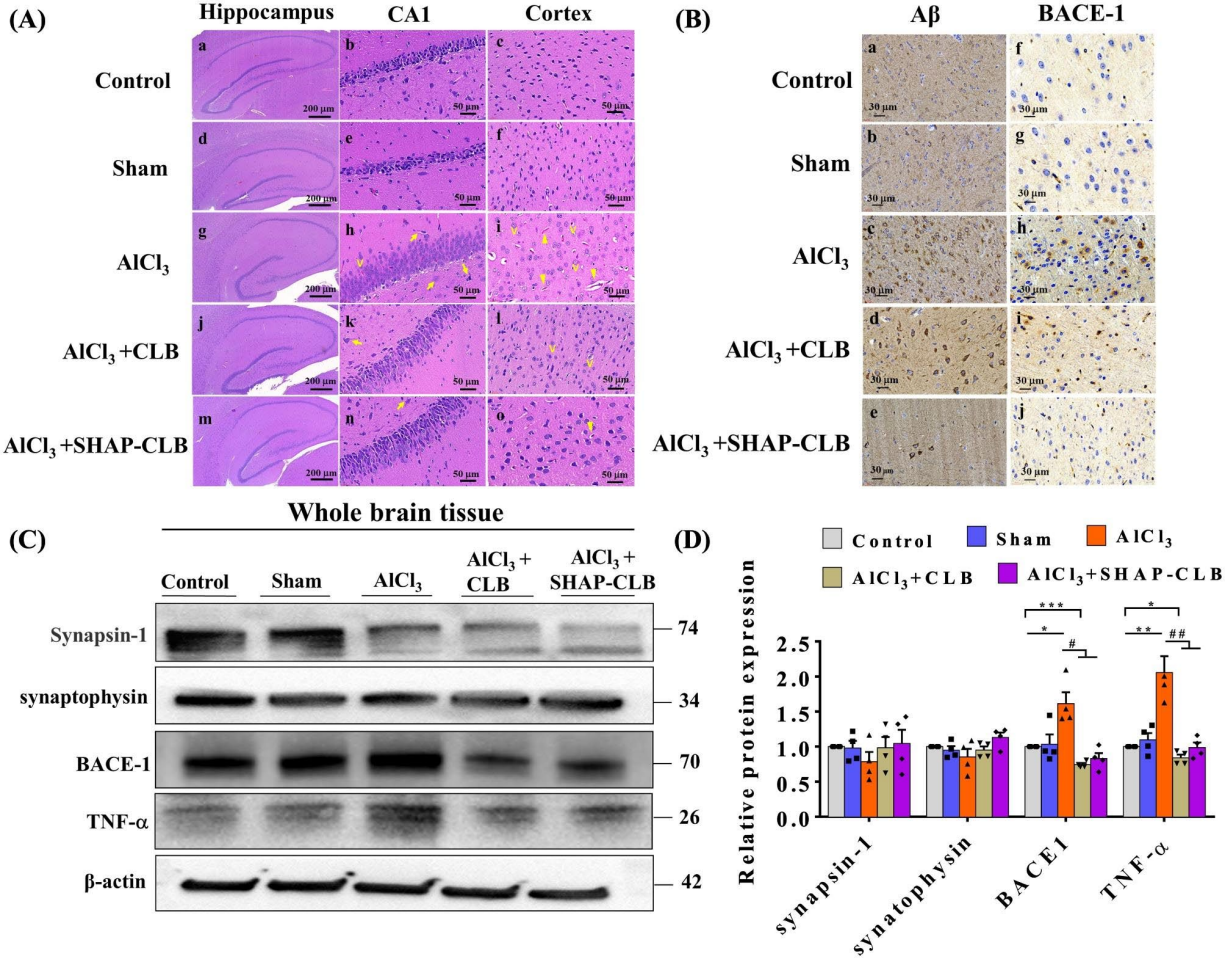
Histological staining of brain sections shows that CLB activates the signaling pathway in the hippocampus of AD rats **(A)** Hematoxylin and eosin (H&E) staining. **(B)** Immunohistochemical staining for BACE-1 and 6E10 (amyloid plaques). Scale bars: 30 μm. **(C)** Western blotting for synapsin-1, synaptophysin, BACE1, and TNF-*α*. **(D)** Quantitative analysis of synapsin-1, synaptophysin, BACE1, and TNF-*α* expression levels. The relative expression levels were normalized against those of β-actin and presented as the ratios of the values in the experimental group to those in the control group (n = 3, *P < 0.05, **P < 0.01 vs. control; #P < 0.05, ##P < 0.01 vs. AlCl_3_).

The structural changes observed in the hippocampus and cortex of the rat brains indicated that the control group animals had normal neuronal morphology in both regions (Fig. 8A). However, rats in the AlCl_3_ group exhibited significant alterations after intraperitoneal administration of AlCl_3_. These changes include an increase in the protruding eosinophilic cytoplasm, pyknotic nuclei, neuronal swelling, shrinkage of pyramidal cells, and dispersed vacuolization, as observed by H&E staining of the rat hippocampus. Furthermore, the AlCl_3_ group exhibited signs of early neuronal injury, focal inflammatory cellular infiltration, and mild cytoplasmic swelling of astrocytes. Some cell nuclei are pyknotic. The brains of rats treated with AlCl_3_ displayed pronounced damage to the hippocampus and cerebral cortex compared with the control group. The most prominent changes observed were cellular atrophy, shrinkage, cellular necrosis, pyknosis, and deeply stained dark nuclei (hyperchromatic cells), as indicated by the arrowhead. Additionally, large cells, mainly multipolar cells, neuronal swelling, chromatolysis, nuclear margination, and vacuolated cells were noted. However, treatment with SHAP-CLB after AlCl_3_ induction improved cerebral damage. Intramuscular administration of SHAP-CLB resulted in mild neuronal toxicity, with a reduced number of eosinophil-stained neurons. The SHAP-CLB-treated groups also exhibited a decrease in pyknotic nuclei and vacuolization, compared with the AlCl_3_ group.

In the hippocampal CA1 region and cortex of the rats subjected to AlCl_3_ treatment, more severe morphological changes were observed in the pyramidal cells of the hippocampal CA1 region. Conversely, the SHAP-CLB group displayed significantly fewer abnormal cells with nuclear condensation. This indicates that SHAP-CLB protects brain tissues from Aβ-induced damage. To determine the pattern of BACE1 localization in the brain of AD, BACE1 and 6E10 immunohistochemistry were performed on sections of the temporal lobe cortex. The increase in BACE1 levels observed in the AlCl_3_ models in correlation with amyloid load demonstrated that the elevation of BACE1 was triggered by Aβ or amyloid plaques (Fig. 8B). BACE1-positive accumulations were present in the cortex and other brain regions with amyloid plaques in the AlCl_3_ group but were completely absent in the normal group. Upon closer examination at higher magnification, the morphologies of BACE1-positive deposits were observed to frequently form ring-like structures with clear cores, while Aβ-positive plaques typically had solid cores. No apparent accumulation was observed in the control or SHAP-CLB groups.

AD is characterized by synaptic loss, and the proteins synapsin-1 and synaptophysin serve as pre- and post-synaptic markers, respectively (Fig. 8C and D). In this study, we examined the expression of synapsin-1 and synaptophysin in the hippocampus of AD rats. Western blot analysis demonstrated a significant reduction in the expression of synapsin-1 and synaptophysin in the hippocampal samples. However, following treatment with CLB and SHAP-CLB, there was a significant increase in the expression of synapsin-1 and synaptophysin, compared with the AD group. These findings suggest that CLB mitigated the loss of synaptic proteins induced by AlCl_3_in vivo. After sacrificing the animals, brain tissue was collected for western blot analysis to assess the levels of synapsin-1, synaptophysin, BACE1, and TNF-α proteins. The results demonstrated that the levels of synapsin-1 and synaptophysin were decreased in the AlCl_3_ model but significantly increased in the treatment group. Considering the role of inflammation in AD, the AlCl_3_ group exhibited significantly higher TNF-α levels. However, the TNF-α levels were significantly reduced in the treatment groups.

## Discussion

This study demonstrated the use of CLB to inhibit the inflammatory reactions caused by Aβ in AD. The blockade of beta-adrenergic receptors by CLB may play a role in AD. To investigate the anti-inflammatory effects of CLB, in this study, we developed synthesized mesoHAP particles as cell-based carriers using a co-precipitation method and with albumin as the foaming agent to facilitate the formation of vesicle-like structures resembling the hydrated and porous networks found in tissues [34]. Surface modification with stearic acid enhanced HAP particle hydrophobicity for easier CLB loading. The infrared spectra of SHAP-CLB proved that they were HAP particles; the XRD pattern and FTIR spectrum data also indicated surface modification with stearic acid [35, 36]. SHAP had rod-like grains that aggregated into particles, forming a mesoporous structure with uniform pore size and adequate porosity. The size of synthesized SHAP-CLB particles ranged from 500 to 1500 nm, suitable for cellular uptake via the endocytic pathway [37]. CLB loading on SHAP involved hydrophobic interactions with the phospholipid hydrocarbon chain and electrostatic interactions with the negative phosphate group.

The BET and TGA results indicate that, as previously suggested by our research [38], the synthesized HAP possesses a larger surface area and porosity, which may accommodate a greater amount of CLB. The loading of CLB occurs through two mechanisms. Firstly, it involves hydrophobic interaction adsorption facilitated by stearic acid surface modification of HAp. Secondly, it involves physical encapsulation within the pores. BET results demonstrate an increase in the surface area of SHAP-CLB, which can be attributed to the CLB adsorbed onto the SHAP surface. TGA curves showed minimal weight loss around 100 °C due to water evaporation. HAP or SHAP exhibited a 13.01% weight loss at 263.05 °C, corresponding to CLB decomposition. The weight loss at 383 °C indicated stearic acid evaporation from the surface. SHAP-CLB achieved approximately 78.06% CLB entrapment efficiency. LCMS and TEM showed effective phagocytosis, and SHAP selectively encapsulated the drug and released it within acidic cellular compartments. SHAP-CLB particles were taken up by cells and trapped in endosomes, which fused with lysosomes (pH 3–5). Within these complexes, SHAP-CLB dissolved, generating high osmotic pressure due to high Ca^2+^ and PO_4_^3−^ concentrations. Eventually, the complex ruptured under osmotic pressure, releasing CLB into the cytoplasm. CLB was then pumped into the intercellular space, reaching nearby blood vessels and circulating to the brain. This controlled release mechanism avoids burst release, which can cause toxicity, particularly during initial treatment days, with symptoms like tachycardia, palpitations, tremors, anxiety, hypokalemia, and hyperglycemia [39]. Therefore, SHAP-CLB nanoparticles enable controlled drug release, crucial for efficient in vivo transport.

The results of the in vitro study indicated the inhibitory effects of CLB on amyloid aggregation. The process of amyloid β aggregation is a critical characteristic of AD and is thought to contribute to the neurodegeneration associated with the condition. This study represents the first report of the remarkable anti-inflammatory effects of CLB on N2a cells exposed to β-amyloid. The blood–brain barrier allows the passage of hydrophobic and small-molecule drugs, such as CLB, through the bloodstream [40]. Therefore, this compound is a promising therapeutic agent against conditions associated with β-amyloid accumulation. However, only a few studies have investigated the pharmacokinetics of multiple CLB doses in rats. Therefore, this study established a specific method that meets the requirements of clinical pharmacokinetic trials. The calibration curve of CLB was linear over the 0.5–200 ng/mL range (linear regression equation: y = 0.01050x + 0.00189; r = 0.99952). The lower limit of quantification for CLB was 0.5 ng/L. In addition, the intracellular and interaction accuracies of CLB were acceptable.

For the in vivo study, the plasma concentration of CLB was measured using LC-MS/MS, and the plasma half-life of CLB was approximately 30 h in rats. Regarding the pharmacokinetics of CLB, a remarkable decrease in clearance was observed at 24 h. The SHAP-CLB group showed stable release until 120 h, which was almost three times longer than that of the free drug. Dynamic monitoring was performed for potential adverse reactions, and no severe adverse reactions were observed.

Animals with AD show decreased neural activity in specific brain regions, particularly the hippocampal CA1 and cortical regions. Our findings also provide new insights into tracking neural abnormalities in animal models of AD using rs-fMRI techniques, suggesting that fALFF analysis using rs-fMRI techniques can serve as an imaging biomarker for measuring aberrant neural activity in animal models of AD. Histopathological analysis revealed Aβ-mediated neuronal damage in hippocampal sections of SD rats, characterized by disorganized neurons, eosinophil-stained cytoplasm, swollen nuclei, and neuronal shrinkage [41]. IHC staining confirmed increased Aβ levels in the hippocampus in the Aβ (1–42) group. Thus, this study also demonstrates the histopathological effects of CLB in AD for the first time. Treatment with SHAP-CLB showed significant protection against Aβ-mediated neuronal damage and plaque formation in the hippocampus. Histological staining indicated that the AlCl_3_-treated hippocampus and many pyknotic nuclei. However, after SHAP-CLB treatment, the cerebral damage improved, neurodegeneration decreased, and Aβ aggregation in the brain decreased. H&E staining of the SHAP-CLB-treated group showed mild neuronal toxicity, with fewer eosinophil-stained neurons and an increased number of healthy neurons with prominent nuclei. Additionally, IHC staining of hippocampal sections from the SHAP-CLB-treated groups demonstrated a protective effect against plaque formation. This analysis supports the hypothesis that SHAP-CLB attenuates the production and accumulation of Aβ in the hippocampus of SD rats. Immunostaining revealed that BACE1 accumulated in ring-like deposits, mirroring the distribution of amyloid plaques. BACE1 is concentrated in the neurons surrounding the Aβ-containing plaque cores in the brains of patients with AD. This study is the first to demonstrate that BACE1 levels increase early and in parallel with amyloid pathology in AD rat models.

CLB demonstrates marked anti-inflammatory properties and modulates microglial activity. Inflammation is considered to be an early event in AD and is believed to play a crucial role in its pathogenesis. NF-κB, a transcription factor, is involved in the activation of various genes associated with inflammation. During neuroinflammation, NF-κB is translocated to the nucleus through the phosphorylation of IκBα, leading to the transcription of pro-inflammatory cytokines and the initiation of an inflammatory response. The current study demonstrated that treatment with CLB alleviated AlCl3-induced AD by modulating the activation of the NF-κB pathway. CLB treatment reduced NF-κB phosphorylation and subsequently lowered the levels of pro-inflammatory cytokines, indicating its potential to inhibit the activation of the NF-κB signaling pathway. The activation of β2AR, due to inhibited NF-κB-dependent gene expression, can reduce the production of pro-inflammatory mediators and promote anti-inflammatory responses in the brain [42]. CLB functions as a β2-adrenergic agonist, and previous studies have reported decreased levels of β2AR and norepinephrine in various regions of AD brains. Activation of the β2AR has been reported to prevent the inhibitory effects of Aβ on long-term potentiation, although the underlying mechanism is unclear [43]. Activation of the β2AR might reduce Aβ accumulation through the upregulation of α-secretase activity and a decrease in APP phosphorylation. Activation of the β2AR enhances amygdala function, and more recently, hippocampal function [44]. Additionally, CLB may enhance memory storage processes and long-term potentiation of downstream targets in the basolateral amygdala, such as the hippocampus, through the involvement of an activity-regulated cytoskeletal protein [45].

If Aβ plays a central role in AD, chronic neuroinflammation serves as a medium that creates a neurotoxic environment, further exacerbating AD complications. Chronic release of pro-inflammatory cytokines, such as TNF-α and IL-1β, can enhance Aβ production by upregulating the expression of APP and promoting tau hyperphosphorylation [46]. The promoter region of BACE-1, which is involved in Aβ production, contains an NFκB binding domain [47]. Stimulation of NFκB by TNF-α and IL-1β can upregulate BACE-1 expression, leading to increased production of neurotoxic Aβ [48]. The reduction of TNF-α level and inhibition of NFκB may contribute to the attenuation of BACE-1 expression, ultimately leading to a decrease in Aβ production. The western blotting analysis demonstrated that the levels of Synapsin-1 and synaptophysin, proteins associated with synaptic function, were reduced in the AlCl_3_-induced AD model. However, in the SHAP-CLB-treated group, Synapsin-1 and synaptophysin levels significantly increased. This indicates that SHAP-CLB treatment can restore synaptic protein levels and improve hippocampal synaptic function. Regarding inflammation, TNF-α levels were significantly higher in the AlCl_3_-induced AD group, suggesting an increased inflammatory response. However, in the groups treated with SHAP-CLB, TNF-α levels were significantly reduced. This indicates that SHAP-CLB treatment exhibits anti-inflammatory effects and can effectively suppress TNF-α production in the hippocampus. These findings suggest that SHAP-CLB treatment can attenuate synaptic dysfunction and reduce inflammation, which are the key pathological features of AD.

The pharmacological synergy and drug mechanism of CLB in AD have been confirmed (Additional File 1), although the exact mechanism of how CLB unravels amyloid plaques is still unclear. The mechanism may be similar to that described in our previous study [49], suggesting that CLB may potentially use its three-dimensional structure to bind with amyloid plaques and impede their self-assembly. Further research is needed to understand the precise action of CLB on amyloid plaques. Additionally, the use of AlCl3-induced AD as the only animal model has limitations, and further studies are required to validate the anti-AD effect of CLB in other AD models.

## Conclusions

The SHAP-CLB system effectively inhibited Aβ self-assembly, protected cells from inflammation-induced damage, improved locomotor activity in rats with induced AD, and reduced inflammation levels in brain tissues. Thus, SHAP-CLB shows promising potential as a therapeutic agent for neurodegenerative disorders.

### Electronic supplementary material

Below is the link to the electronic supplementary material.

**Additional file 1**: Pharmacological synergy and proposed mechanism of action of clenbuterol in Alzheimer’s disease

## Data Availability

The datasets supporting the conclusions of this article are included within the article and its additional files.

## Abbreviations

AD: Alzheimer’s disease
Aβ: Amyloid-β
SHAP: Synthesized mesoporous hydroxyapatite
CLB: Clenbuterol
SHAP-CLB: CLB-loaded SHAP
HAP: Hydroxyapatite
APP: Amyloid precursor protein
ROS: Reactive oxygen species
IFNγ: Interferon γ, TNFα, tumor necrosis factorα
IL-1β: Interleukin 1β
IL-6: Interleukin 6
AChEIs: Acetylcholinesterase inhibitors
FDA: Food and Drug Administration CLB, Clenbuterol
β2AR: Beta 2 adrenergic receptor
BMMs: Bone marrow-derived macrophages
LPS: Liposaccharide
mesoHAP: Mesoporous HAP
XRD: X-ray diffraction
SEM: Scanning electron microscopy
BET: Brunauer–Emmett–Teller
FTIR: Fourier transform infrared spectroscopy
TGA: thermogravimetric analysis
WST-1: Water-soluble tetrazolium
TEM: Transmission electron microscopy
MWM: Morris Water Maze
SD: Sprague-Dawley
LC-MS/MS: Liquid Chromatography Triple Quadrupole Mass Spectrometry
DMSO: Dimethyl sulfoxide
PBS: Phosphate buffered saline
fMRI: Functional magnetic resonance imaging
RARE: Rapid acquisition and relaxation enhancement
SPM: Statistical parameter mapping
AFNI: Analysis of functional neuroimaging
fALFF: Fractional amplitude of low-frequency fluctuations
ROI: Region of interest
QC: Quality control, BACE1, β-site amyloid precursor protein cleaving enzyme-1
H&E: Hematoxylin and eosin
IHC: Immunohistochemical
TBS: Tris-buffered saline
DLS: Dynamic light scattering
JCPDS: Joint Committee on Powder Diffraction Standards
BJH: Barrett–Joyner–Halenda
rs-fMRI: Resting state fMRI
