# Supplementary material for: Controlled release of Clenbuterol from a hydroxyapatite carrier for the treatment of Alzheimer’s Disease
Source: Biomater Res. 2023 Oct 5;27:98. doi: 10.1186/s40824-023-00432-4 (PMC10557233; doi:10.1186/s40824-023-00432-4)
Supplement: Supplementary file 1 — Additional file 1: Pharmacological synergy and proposed mechanism of action of clenbuterol in Alzheimer’s disease [file 40824_2023_432_MOESM1_ESM.docx]

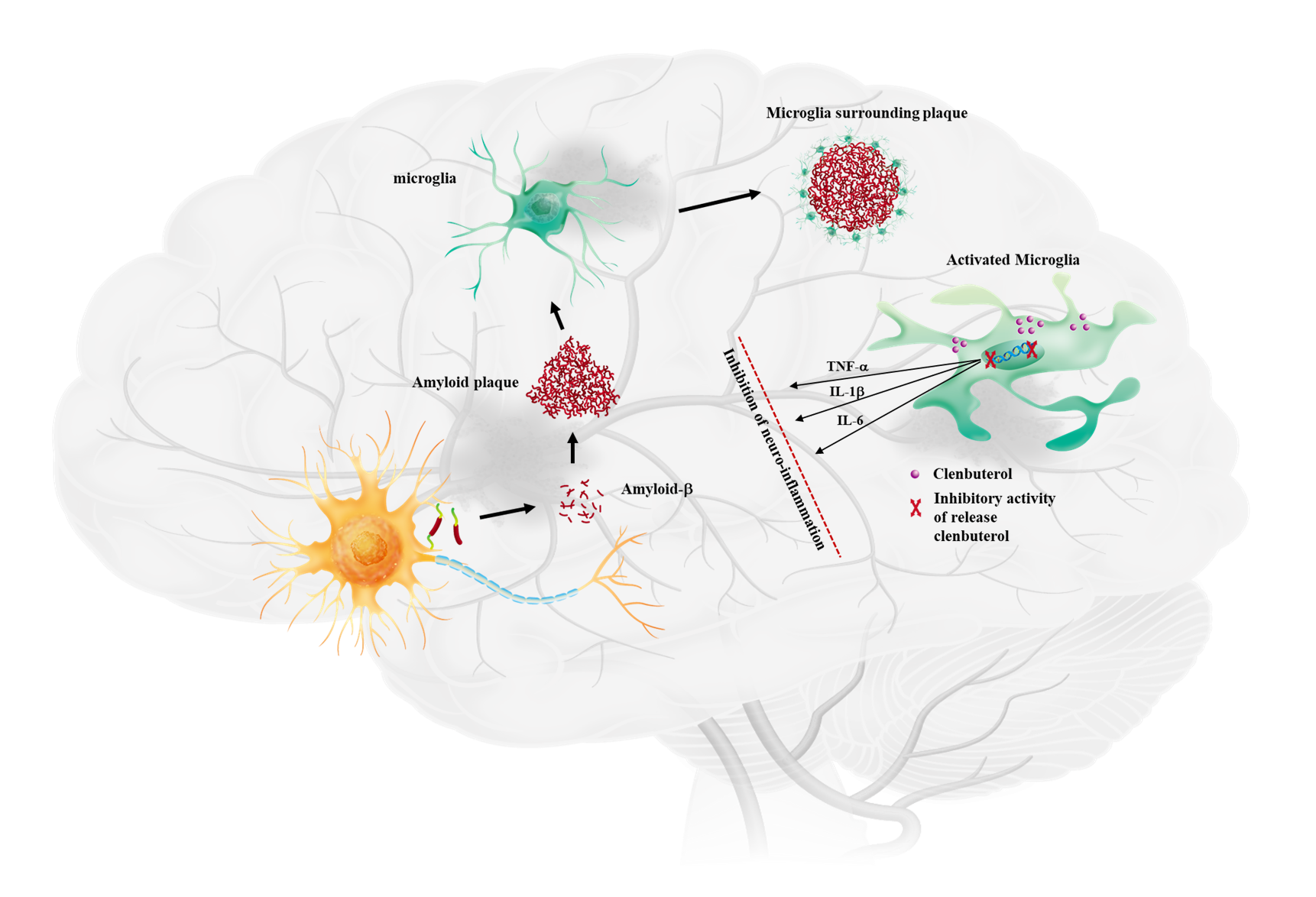


**Additional File 1.** Pharmacological synergy and proposed mechanism of action of clenbuterol in Alzheimer's disease
